# Concurrent Gastrointestinal Perforation and Cardiopulmonary Arrest in Toxic Epidermal Necrolysis: A Case of Profound Multi-organ Involvement

**DOI:** 10.7759/cureus.108665

**Published:** 2026-05-11

**Authors:** Sheikha Alketbi, Amani Abdulla AlFalasi, Alia Galadari

**Affiliations:** 1 Dermatology, Rashid Hospital, Dubai Health, Dubai, ARE; 2 Dermatology, Mohammed Bin Rashid University of Medicine and Health Sciences, Dubai, ARE

**Keywords:** cardiopulmonary arrest, colonic perforation, gastrointestinal perforation, multiorgan failure, severe cutaneous adverse reaction, toxic epidermal necrolysis

## Abstract

Toxic epidermal necrolysis (TEN) is a serious, life-threatening cutaneous adverse reaction whose prevalence is widespread epidermal necrosis and detachment, mostly initiated by an adverse drug reaction. Muco-cutaneous manifestation is well-known, but severe systemic complications, specifically gastrointestinal complications and cardiac events, are extremely uncommon, with few cases reported in the literature.

This case describes a 27-year-old man who was diagnosed with TEN after taking ibuprofen. He had colonic perforation and cardiopulmonary arrest despite systemic therapies and a multidisciplinary approach.

The case study shows that even though TEN is associated with poor prognosis, there are rare serious complications, which are likely to be mediated in this case by the excessive systemic inflammatory response and coagulopathy due to sepsis, and have detrimental outcomes. These unusual complications must be detected early and dealt with aggressively to enhance patient survival in severe TEN.

## Introduction

Toxic epidermal necrolysis (TEN) is a rare, life-threatening, severe cutaneous adverse reaction characterized by widespread epidermal necrosis and detachment, most commonly triggered by medications [1]. Although mucocutaneous involvement is the hallmark of the disease, systemic complications can occur and significantly contribute to morbidity and mortality.

Gastrointestinal involvement in TEN is uncommon, and intestinal perforation is an exceptionally rare but severe complication with limited cases reported in the literature [2,3]. Cardiopulmonary complications are less well defined but may occur in the setting of severe systemic inflammation and sepsis.

We report a rare case of TEN complicated by colonic perforation and cardiopulmonary arrest, highlighting the potential for profound multi-organ involvement and the importance of early recognition and multidisciplinary management.

## Case presentation

A previously healthy 27-year-old Pakistani male presented with a four-day history of fever (39.2°C) and sore throat. A generalized rash developed one day after self-administering ibuprofen 400 mg for symptom relief. Concurrently, he experienced significant mucosal involvement affecting oral, ocular, and genital mucosae, manifesting as an inability to open his eyes, dysphagia, and dysuria. The rash initially appeared at the left antecubital fossa, prompting evaluation by a family physician at a local clinic, where he received intravenous paracetamol for symptomatic fever management and oral prednisolone 30 mg daily for presumed allergic or inflammatory skin eruption for two to three days. Despite treatment, the rash rapidly progressed with severe multi-mucosal involvement. This was his first episode of such an eruption with no history of regular medications or previous drug reactions.

**Figure 1:**
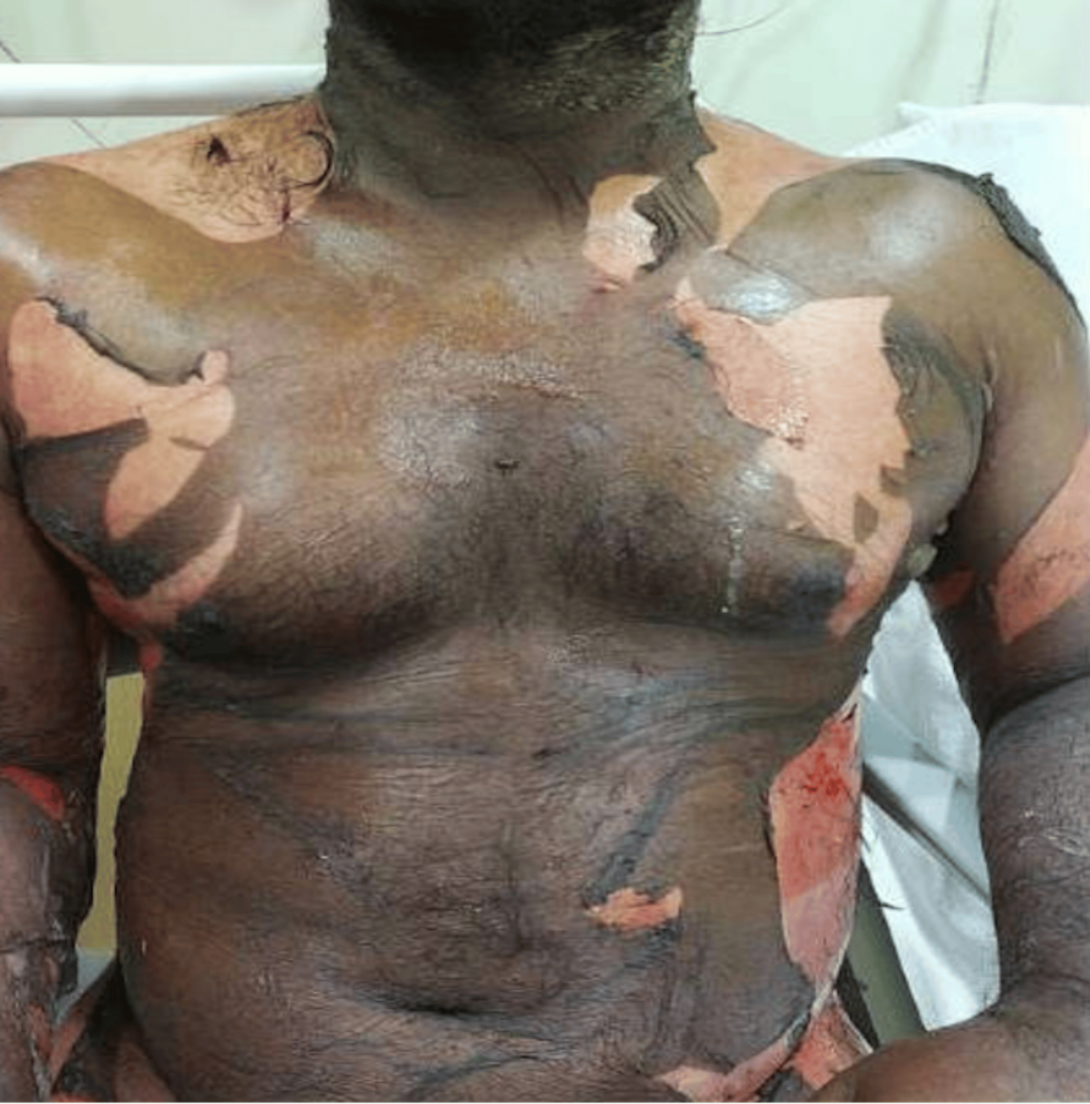
Widespread epidermal detachment and peeling on the trunk and upper extremities at presentation.

Upon presentation to our emergency department, the patient exhibited diffuse dusky red macular eruptions coalescing into plaques with widespread epidermal detachment affecting approximately 90% of his total body surface area (Figure 1).

**Figure 2:**
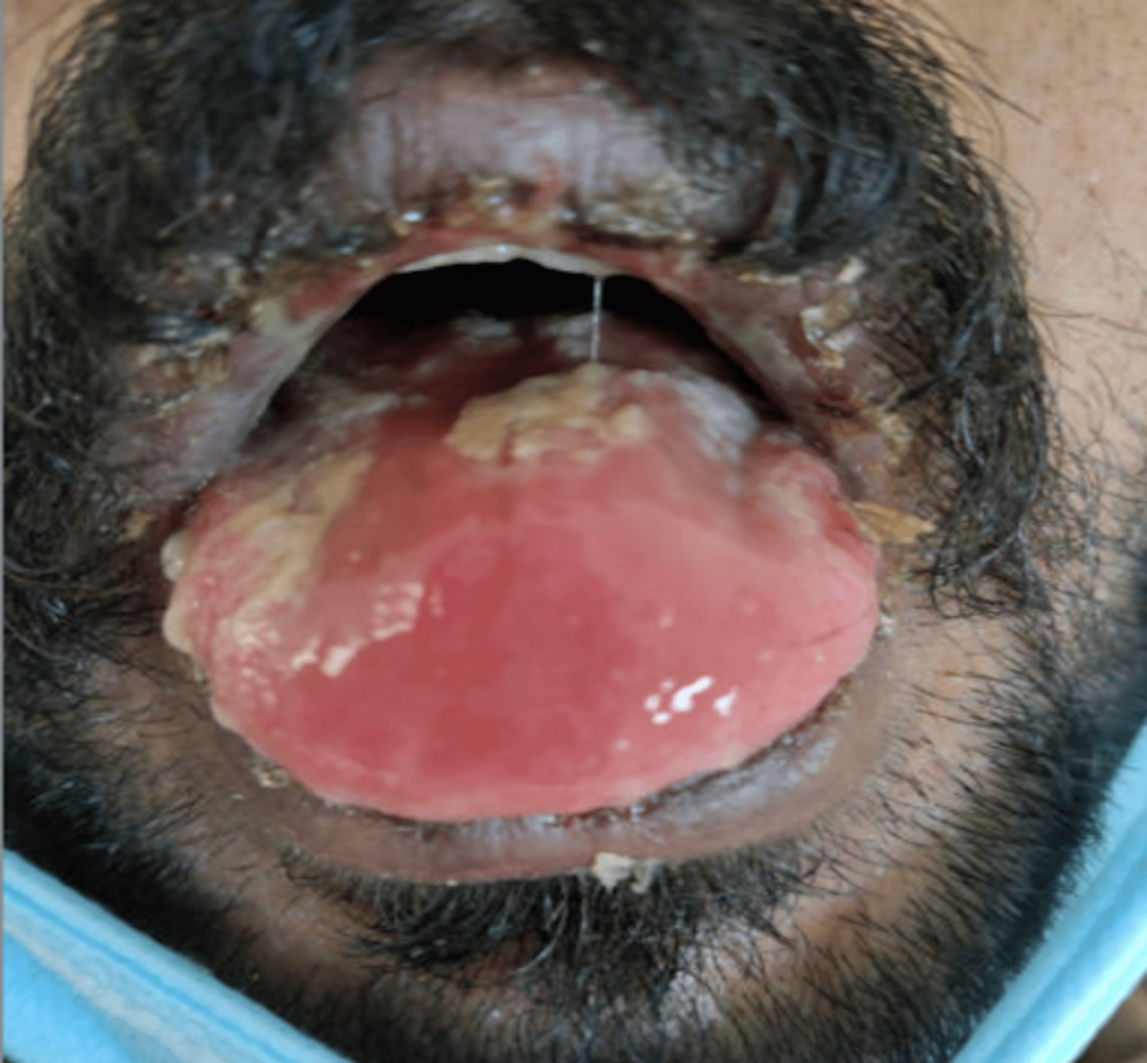
Severe oral mucosal involvement with extensive ulceration and sloughing at presentation.

Additionally, he had painful oral and genital mucosal ulcerations and severe ocular conjunctivitis with conjunctival injection and erosions (Figure 2). The Severity-of-Illness Score for Toxic Epidermal Necrolysis (SCORTEN) on admission was calculated as 3 [4-6], indicating an estimated mortality rate of approximately 35%. The SCORTEN score was used for clinical prognostic assessment only, and no part of the original scoring system or table has been reproduced.

**Figure 3:**
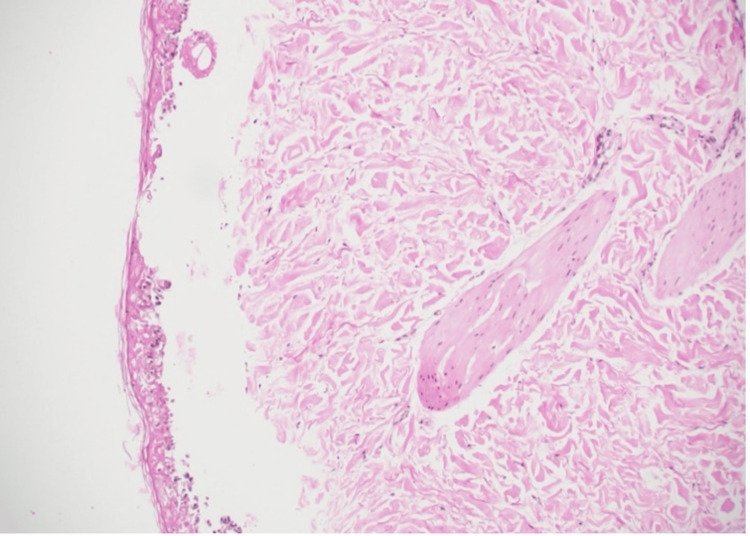
Skin biopsy histopathology showing full-thickness epidermal necrosis with dermo-epidermal separation and a basket-weave stratum corneum (hematoxylin and eosin stain, ×20 magnification).

Skin punch biopsy confirmed the clinical diagnosis of TEN. Histopathological examination revealed full-thickness epidermal necrosis with dermo-epidermal separation (Figure 3). Direct immunofluorescence demonstrated amorphous necrotic epidermis separating from dermis, with negative staining for IgM, IgG, and IgA. Diffuse C3 positivity was observed in small dermal blood vessel walls, and fibrin deposition was positive within the necrotic epidermis.

The patient was admitted to the burn unit for specialized management, including meticulous wound care with non-adhesive dressings, emollients, and stringent infection control measures. A multidisciplinary approach was employed involving dermatology, critical care, infectious diseases, and surgical teams. He received etanercept 50 mg on admission and day 8, along with intravenous immunoglobulin (IVIG) at 1 g/kg for three days. Initial treatment with intravenous hydrocortisone 100 mg twice daily was supplemented with topical corticosteroids and antibiotics.

During hospitalization, the patient experienced progressive deterioration with inflammatory markers rising significantly. Procalcitonin levels reached 9.4 ng/mL alongside signs of acute kidney injury. Laboratory findings indicated disseminated intravascular coagulation with significant coagulation defects, elevated D-dimer, abnormal liver function tests, and hypoalbuminemia. High-sensitivity troponin I/T levels were elevated, and both sputum and blood cultures were positive for methicillin-resistant *Staphylococcus aureus*. Ferritin levels reached 1417 ng/mL with significant electrolyte abnormalities. He developed anemia (Hb 7.1 g/dL), and wound cultures were positive for *Pseudomonas *infection. Laboratory findings are summarized in Table 1.

**Table 1:** Summary of laboratory values shown were obtained during the period of clinical deterioration. MRSA: methicillin-resistant *Staphylococcus aureus*. "Abnormal" indicates values outside the reference range.

Parameter	Result	Reference range
Procalcitonin	9.4 ng/mL	<0.05 ng/mL
Hemoglobin (Hb)	7.1 g/dL	13–17 g/dL
Ferritin	1417 ng/mL	30–400 ng/mL
Lactate	6.8 mmol/L	0.5–2.2 mmol/L
D-dimer	Elevated	<0.5 µg/mL
Liver function tests	Abnormal	Within normal limits
Albumin	Low (hypoalbuminemia)	3.5–5.0 g/dL
Troponin	Elevated	<0.04 ng/mL
Electrolytes	Abnormal	Within normal limits
Blood culture	MRSA positive	Negative
Sputum culture	MRSA positive	Negative
Wound culture	Pseudomonas positive	Negative

On hospital day 30, the patient suddenly collapsed, requiring cardiopulmonary resuscitation. The patient was initially managed in the burn unit with multidisciplinary care and was later transferred to the general ward following partial clinical stabilization and improvement in skin re-epithelialization. In the days preceding the deterioration, he complained of progressive abdominal pain with worsening clinical status. On hospital day 30, he suddenly collapsed, requiring cardiopulmonary resuscitation. He was subsequently intubated and transferred to the intensive care unit. Chest radiography performed following resuscitation incidentally demonstrated pneumoperitoneum.

Emergency exploratory laparotomy revealed a single 2 mm microperforation on the anterior wall of the mid-transverse colon, which was primarily repaired. Marked dilatation of the large bowel, particularly involving the transverse colon, was noted intraoperatively.

Laboratory investigations obtained following the cardiopulmonary arrest demonstrated severe metabolic acidosis with lactate levels of 6.8 mmol/L, indicating profound tissue hypoperfusion. The patient’s Glasgow Coma Scale score was 3/15 with fixed dilated pupils, consistent with severe hypoxic-ischemic brain injury following cardiac arrest.

**Figure 4:**
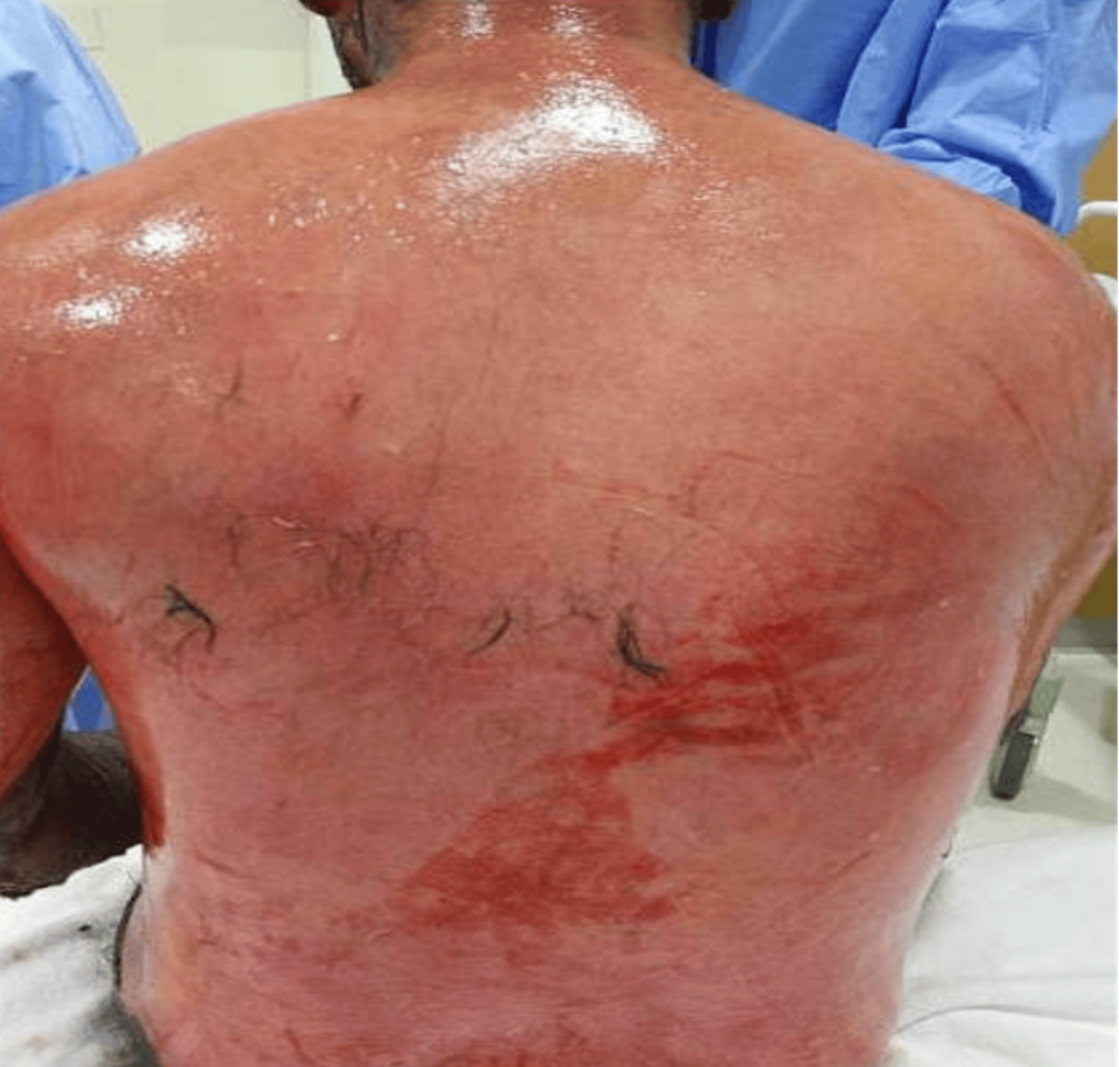
Significant re-epithelialization and healing observed on the patient's back during recovery.

**Figure 5:**
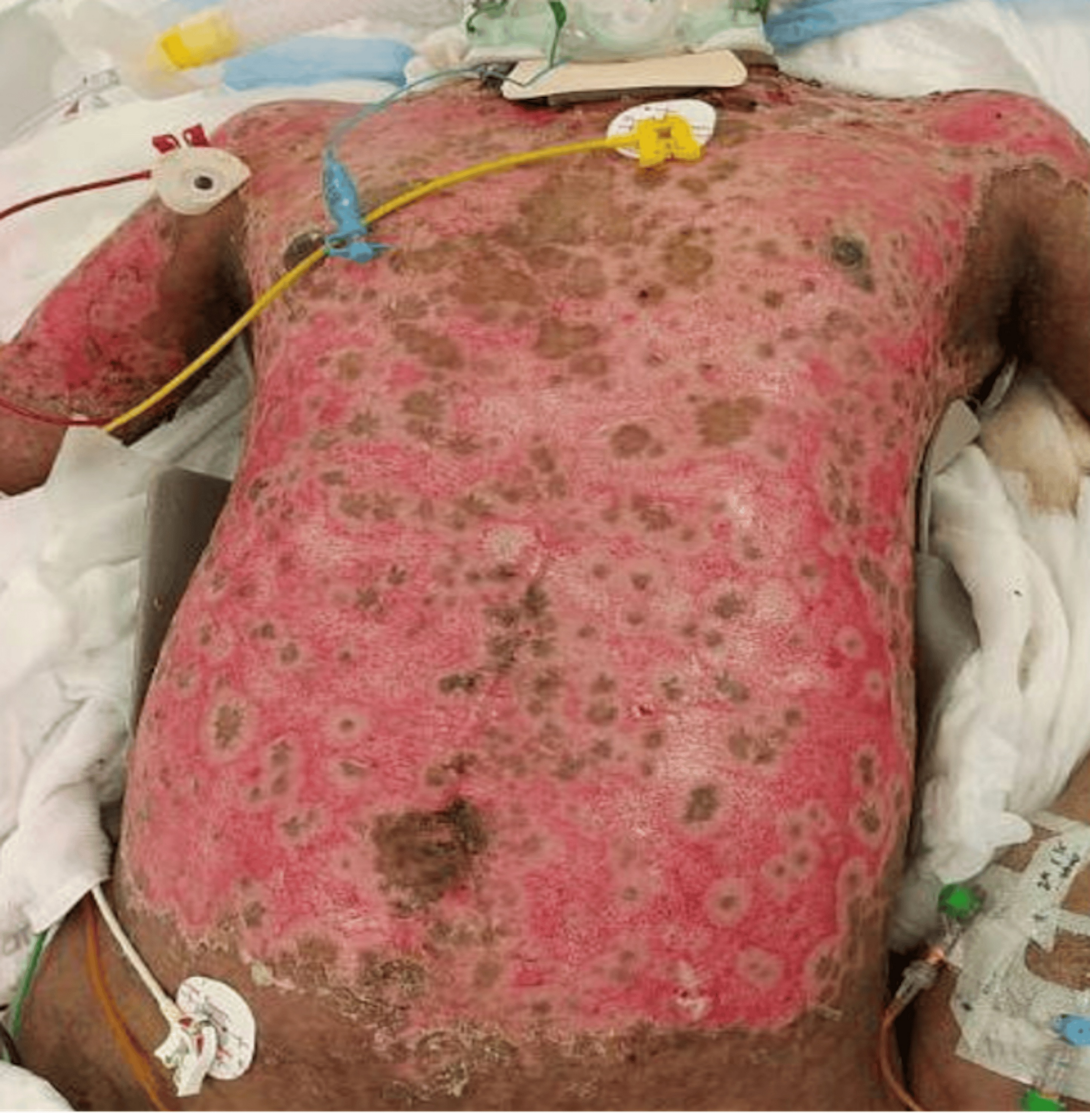
Extensive body surface area involvement and ongoing skin changes during the intensive care phase of toxic epidermal necrolysis.

Prior to the acute deterioration, significant improvement in the cutaneous lesions had been observed, with progressive drying and re-epithelialization of trunk lesions and near-complete resolution of facial and extremity involvement following meticulous wound care (Figures 4, 5).

The clinical course was consistent with severe sepsis complicated by disseminated intravascular coagulation and septic shock, leading to multi-organ dysfunction. The intestinal perforation was considered a possible complication occurring in the setting of severe TEN-associated systemic inflammation, disseminated intravascular coagulation, and septic shock.

## Discussion

This case demonstrates the severity of TEN and its potential for rare systemic complications. TEN is a life-threatening, predominantly drug-induced mucocutaneous reaction associated with high morbidity and mortality [1]. While cutaneous and mucosal involvement are typical, internal organ complications such as gastrointestinal involvement are uncommon [3-5,7].

Neurological deterioration following cardiac arrest, reflected by a low Glasgow Coma Scale score, suggested severe hypoxic-ischemic brain injury [2].

Differentiation from mimickers such as staphylococcal scalded skin syndrome is important [8].

The SCORTEN score is a useful prognostic tool, with higher scores associated with increased mortality [6,9]. Our patient’s score of 3 was consistent with his clinical course. The presence of severe sepsis and multi-organ dysfunction further worsened the prognosis [1,3].

Histopathology and direct immunofluorescence helped confirm the diagnosis and exclude other conditions [10].

Management of TEN is primarily supportive and requires a multidisciplinary approach, including meticulous wound care, infection prevention, and critical care support [11].

Gastrointestinal involvement in TEN is uncommon but has been reported in severe cases, particularly in association with extensive mucosal injury, systemic inflammation, sepsis, and multi-organ dysfunction [3,7]. In the present case, the exact mechanism underlying the colonic perforation remains uncertain and was likely multifactorial. Possible contributing factors include TEN-associated mucosal injury, disseminated intravascular coagulation, septic shock, intestinal ischemia, and critical illness-related bowel dysfunction. Furthermore, as pneumoperitoneum was identified following cardiopulmonary resuscitation, a contributory role of resuscitative efforts cannot be entirely excluded, although CPR-related gastrointestinal injury is considered uncommon [12].

Histopathological examination of intestinal tissue was not available; therefore, a definitive causal relationship between TEN and the bowel perforation could not be established. This represents an important limitation of the present report.

This case underscores the importance of recognizing atypical systemic complications in severe TEN, particularly in patients with rapid clinical deterioration or sepsis.

## Conclusions

Toxic epidermal necrolysis is a serious multisystem condition that can have life-threatening internal organ complications that are uncharacteristic of its typical mucocutaneous manifestations. This case illustrates the relevance of a high index of suspicion in the case of atypical systemic complications in patients with clinical deterioration, such as unexplained sepsis or refractory metabolic abnormalities. These life-threatening complications as gastrointestinal perforation need early identification and multidisciplinary care so that the best possible results are achieved in severe TEN.
